# Mate Choice in Double-Breeding Female Great Tits (*Parus Major*): Good Males or Compatible Males

**DOI:** 10.3390/ani11010140

**Published:** 2021-01-11

**Authors:** Qianxi Fan, Mingju E, Yusheng Wei, Wei Sun, Haitao Wang

**Affiliations:** 1 Jilin Engineering Laboratory for Avian Ecology and Conservation Genetics, School of Life Sciences, Northeast Normal University, Renmin Street 5268, Changchun 130024, China; fanqx972@nenu.edu.cn (Q.F.); emj640@nenu.edu.cn (M.E); weiys677@nenu.edu.cn (Y.W.); 2 Key Laboratory for Vegetation Ecology, Ministry of Education, Institute of Grassland Science, Northeast Normal University, Renmin Street 5268, Changchun 130024, China; 3 School of Life Sciences, Changchun Normal University, 677 Changjibei Road, Changchun 130032, China; 4 Jilin Provincial Key Laboratory of Animal Resource Conservation and Utilization, School of Life Sciences, Northeast Normal University, Renmin Street 5268, Changchun 130024, China

**Keywords:** double breeding, mate choice, compatible genes, good genes

## Abstract

**Simple Summary:**

Double breeding is a common reproductive strategy among temperate passerines to increase annual fecundity. To produce two clutches in the same breeding season and to ensure offspring quality, choosing a good mate is important for females. Uncovering the method used in social mate choice for genetic benefits adopted by double-breeding females would provide a better understanding of the life history and rules of female choice. In the present study, we tested the effects of the date of the first egg of the first brood and of female quality on double breeding, and good genes and genetic compatibility hypotheses on mate choice for double breeding female great tits (*Parus major*) in a population breeding inside nest boxes of Zuojia Natural Reserve in northeast China. The date of the first egg of the first brood did not affect initiation of a second brood, and female individual heterozygosity slightly influenced initiation of a second breeding. Female great tits choose males with both compatible genes and good genes in double-breeding mating. Double-breeding females prefer males with large breast stripes, high heterozygosity, and lower relatedness. The number of offspring of the first clutch did not affect the pairing status of male great tits in double breeding. The genetic quality of offspring from double-breeding pairs was higher than that of those from single-breeding pairs (higher heterozygosity and lower individual *F*).

**Abstract:**

Producing two broods within the same season may be a good strategy by which short-lived species can maximize reproductive success. To produce two clutches in the same breeding season and to ensure offspring quality, choosing a good mate is important for females. Previous studies on double breeding focused on the associated influencing factors, and few studies examined how females choose social mates. Good genes and genetic compatibility are the two main hypotheses of the genetic benefit that females obtain from choosing mates. Uncovering the method used in mate choice for genetic benefits adopted by double-breeding females would provide a better understanding of the life history and rules of female choice. The great tit is an optionally double-breeding species in temperate-latitude populations. Here, we used a dataset for a Chinese population monitored between 2014 and 2016 to test two hypotheses on double-breeding female mate choice. A total of 30.1% of the breeding pairs initiated second breeding attempts, always remating with the same mate. The date of the first egg of the first brood did not affect initiation of a second brood, and female individual heterozygosity slightly influenced initiation of a second breeding. Female great tits choose males with both compatible genes and good genes in double-breeding mating. Double-breeding females prefer males with large breast stripes, high heterozygosity, and lower relatedness, while tarsus length, repertoire size, and individual *F* are not the main factors considered by females when selecting males for double breeding. The number of offspring of the first clutch did not affect the pairing status of male great tits in double breeding. The genetic quality of offspring from double-breeding pairs was higher than that of those from single-breeding pairs (higher heterozygosity and lower individual *F*). Taken together, our results showed that double breeding female great tits adopt multiple methods for genetic benefits to choose mates.

## 1. Introduction

Mate choice is the most important part of the reproductive process. Reproductive individuals are predicted to choose their mates based on the potential direct benefits (“good parent”) or indirect benefits (“good genes”) that they provide [1]. This choice can manifest in behavioral patterns and eventually in genetic patterns [2,3]. Mate choice for genetic benefits is an important component of many breeding systems [4]. Females are generally choosier than males because they invariably invest more in their gametes [5]. Female choice for genetic benefit has been extensively researched, and there are two main hypotheses [4,6]. The good genes hypothesis states that, when mating with genetically superior males, females may produce offspring with higher fitness and may gain additive genetic benefits [7,8], and the compatibility hypothesis postulates that mating with compatible males could increase offspring genetic diversity and could reduce deleterious recessive allele expression [2,5]. Furthermore, heterozygosity preferences may be considered a specific example of a “good genes” indicator trait [9,10]. These two hypotheses are not mutually exclusive, and females may use both methods to choose mates.

Females mate with males who contribute good or compatible genes to their offspring, thereby increasing the genetic quality of their offspring [4]. Good genes that can increase fitness independently of the remainder of the genome show that additive genetic variance can be conveyed by condition-dependent traits [11]. Females may using these traits to choose males with “good genes” [12] because offspring from these mates are more likely to survive and are more attractive [13,14].

Ornaments, body size, or displays can be regarded as honest signals of an individual’s intrinsic quality [15]; females may indirectly assess male quality through their characteristics. Tarsus length is an important characteristic of birds that may affect female choice and is considered an indicator of the genetic quality of males [16]. Some ornaments are repeatable and heritable, such as the black breast stripe of the great tit (*Parus major*) [14] that is thought to be an indicator of individual quality and to play an important role in mate selection [14,17,18,19]. Songs are a primary target of sexual selection in birds, and females may choose males with high-quality songs because it provides information about male quality [20,21], reflecting the age, survival success, and condition of the male [21]. In particular, dawn songs have been shown to be especially important in mate choice [22,23].

Individual heterozygosity may in itself be beneficial in terms of gaining mating opportunities [24]. In mate choice, individuals may prefer heterozygous partners, as heterozygosity increases, the number of different genes inherited by offspring is also likely to increase [5,24], and they may produce more heterozygous offspring [25,26]. Heterozygosity is also generally beneficial to individuals because it may mask lethal and sublethal genes. The fitness of the offspring of heterozygous males is greater than the population mean fitness [24]. In addition, heterozygosity appears to underlie the superiority of males with respect to developmental stability, immunocompetence, parasite resistance, general condition, and attractiveness [24,27,28] and has been shown to influence investment to a greater extent in their offspring [9,29]. Genetic characteristics of individuals are important for reproductive success [9,27,29]. In addition to heterozygosity, the individual inbreeding coefficient (individual *F*) is also an important indicator of individual genetic quality.

Genetic compatibility is often described as the degree of genetic dissimilarity between potential mates [5,30]. In contrast with “good genes”, compatible genes increase fitness when paired with a specific genotype and show nonadditive genetic variance [5]. In these effects, the genotypes of both parents determine the genetic quality of progeny, and thus different pairings rather than the simple selection of the most vigorous males are required to increase offspring fitness [4]. Parental genetic similarity is important in mate choice, as reproduction between closely related individuals often reduces offspring fitness, likely because of homozygosity and the expression of deleterious recessive alleles, which in turn induce decreased adaptability to changing environments [31,32]. This effect is generally referred to as “inbreeding depression”. Therefore, to increase their fitness, individuals can produce heterozygous and therefore “fitter” offspring by finding an unrelated or genetically dissimilar mate [5,33], thereby avoiding the negative effects of inbreeding [33,34].

Individuals can adjust their annual fecundity by changing either the number of offspring per breeding attempt or the number of breeding attempts per season [35,36]. Producing two annual broods within the same season may be a good strategy for short-lived species to maximize their annual and lifetime reproductive success. However, not all individuals in most species or populations reproduce twice because many factors influencing the decision to double brood, for instance, the timing of the first clutch [37,38], individual quality [38,39], environmental factors [36,40], the number of fledglings in the first clutch [35,41], etc. Many studies have reported the timing of the first brood and female quality to be the main factors for double breeding. Early breeders are more likely to initiated second clutches because breeding early allows females to extend the length of the breeding season [35,38]. Importantly, the differences among early and late brooding breeders in terms of their own quality (quality hypothesis) [41] and the timing of breeding are often determined by the quality of both male and female parents [42,43,44]; in general, better-quality birds tend to breed earlier. Most previous studies on individual quality have focused on the effects of female quality on double breeding [45] and have rarely considered male parents in double breeding [46]. This is likely because the females have already made the behavioral decision to double breed when brooding their first clutches [47] and are assumed to control initiation of the second breeding. However, most passerine birds are socially monogamous with biparental care, and males may substantially affect reproductive success [48,49]; moreover, male are also mainly responsible for post-fledging care of the first brood [50]. Therefore, a high-quality male may reduce female reproductive efforts and the female is more likely to initiate a second brood.

The great tit is an optionally double-brooded, socially monogamous with biparental care species [51]; females initiate second clutches by remating with the same male. In order to produce two clutches in the same breeding season, it may be important for females to choose a high-quality mate. Previous studies of great tits on double breeding have focused on the influencing factors, such as the timing [40,41,45], clutch size [41], and parental investment [52] of the first broods, but there have been few studies on how females choose social mates. Here, we aimed (i) to assess whether female quality affects initiation of the second brood; (ii) to examine which patterns female great tits use in social mate choice for double breeding; (iii) to identify which male traits females prefer when choosing social mates for double breeding; and (iv) to determine whether gene quality differs between offspring of double- and single-breeding pairs. In this study, we considered tarsus length, breast stripe size, the individual inbreeding coefficient, individual heterozygosity [10,16], and repertoire size as indicators of good genes and pairwise relatedness as an indicator of the genetic compatibility of mates [16].

## 2. Materials and Methods

### 2.1. Ethics Statement

This study conformed to the guidelines for the care and use of experimental animals established by the Ministry of Science and Technology of the People’s Republic of China (approval number: 2006-398). Experimental procedures were permitted by National Animal Research Authority in Northeast Normal University (approval number: NENU-20080416).

### 2.2. Study Area and Data Collection

The study site has an elevation ranging from 200 to 530 m and occurs where the hill region of the Chang-bai Mountains transitions to the plains. The forest in the area is secondary growth deciduous woodland, and the dominant tree species include *Quercus mongolica, Tilia mandshurica, Betula davurica, Salix pierrotii, Fraxinus mandshurica, Sophora japonica, Populus davidiana*, and *Ulmus japonica*. Since 1986, our team has used the Zuojia Nature Reserve as a field base for the study of bird ecology and has performed much research [53], and thus, great tits readily breed in the nest boxes in this area. The study plot contains approximately 450 nest boxes placed approximately 3–4 m above the ground and separated by 30–50 m. The average inner diameter of the wooden nest box was 12 cm × 12 cm × 25 cm; the average horizontal diameter of the entrance hole was 5 cm. The entrance hole was opened at 1/3 of the front wall of the nest box. All nest boxes were the same size. All nest boxes were checked every 3 days from late March until mid-July each year (2014–2016). The intervals of visits to the occupied nest boxes varied depending on the breeding status to minimize disturbance and to optimize the accuracy of information. We recorded the first egg-laying date of each breeder (April 1st was defined as 1 each year, i.e., April 1 = day 1). Adults were captured no earlier than 7 days after the nestlings hatched, either with mist nets or the baffle method, and then were numbered with metal rings.

### 2.3. Morphological Measurements

From each captured bird, we took standard measurements of tarsus length and breast stripe width to the nearest 0.1 mm. We used tarsus length as an indicator of body size [54]. The breast black stripe is an important sexually dimorphic trait of the great tit, and its size may reflect male attractiveness and social status [27,55]. The size of the breast black stripe was measured following Järvi and Bakken (1984) and Poeysae (1988) (the width at the level of the clavicle) [56,57]. It is well known that measuring the size of the black stripe from digital photographs and analyzing them using a program has higher repeatability and accuracy [58,59]. However, we did not have photographs. To increase repeatability [60], we measured the breast black stripe width of each individual′s 3 times and used the average value as its size [19].

### 2.4. Genetic Analysis

Adults were bled by brachial venipuncture, and blood samples (about 30 μL) were stored in 1.5-mL centrifuge tubes with 100% ethanol. For total DNA extraction from blood, we used standard phenol-chloroform. We used a NanoDrop spectrophotometer (Thermo Scientific Inc., Waltham, MA, USA) to quantified DNA concentration and diluted with TE (Tris and Ethylene Diamine Tetraacetic Acid) buffer to >30 ng. We selected the 14 most used loci in previous publications as candidate microsatellite markers (Appendix A [61,62]. Amplifications were conducted in 20-μL reaction volumes. The polymerase chain reaction (PCR) conditions were as follows: 9 min denaturing at 95 °C followed by 35 cycles of 30 s at 94 °C, 40 s at the annealing temperature (Appendix A), and 30 s at 72 °C, ending with a 8-min final elongation step at 72 °C. PCR products were run on an ABI PRISM 3100 Genetic Analyzer (Applied Biosystems, Foster City, CA, USA). Candidate microsatellite markers tested for null alleles and deviations from the Hardy–Weinberg equilibrium (HWE) using CERVUS 3.0 [63] and for linkage disequilibrium between loci using FSTAT [64] (dataset only comprised all adult birds). Microsatellite loci that showed significant departure from Hardy–Weinberg equilibrium or for which linkage disequilibrium was detected were excluded (6 of the 14 loci were eliminated). All null allele frequencies were below 0.20 [65]. Eight loci were employed to estimate genetic relatedness, the inbreeding coefficient, and heterozygosity. Pairwise relatedness, a measure of genetic similarity between individuals, was estimated using COANCESTRY [66] (the dataset only comprised paired adult birds from 2014, 2015, and 2016). The inbreeding coefficient (*F*) was estimated using the method of Ritland (1996) [67] by the program COANCESTRY (the dataset comprised all adults and offspring). Heterozygosity scores were calculated using an Excel macro written by W. Amos [32,68] (the dataset comprised adult and offspring).

### 2.5. Acoustic Recordings and Measurements

We recorded male dawn songs during the egg-laying period of their mates, and the songs were recorded no later than 3:30 a.m. We hid in the bushes near the nest boxes to record the male dawn song to prevent interference with singing. We recorded as many male dawn songs as possible at every nest we found. During breeding season, each male has its own territory; with dawn songs, they generally use the same singing positions. In order to ensure that we recorded males from particular nests, we observed the male singing positions for 1 to 2 days before recording, although we did not mark the birds individually. The most important is that the males usually sing close to their nest box. These recordings were made only during the 1st brood.

A TASCAM HD-P2 portable digital recorder (TEAC Corporation, Tokyo, Japan) and a Sennheiser MKH P48 external directional microphone (Sennheiser electronic GmbH & Co., KG, Wedemark, Germany) were used for recording. We used Avisoft-SASLab Pro version 5.2.10 (Avisoft, Berlin, Germany) to analyze the recordings. We measured the repertoire size of all the dawn songs of one day. We defined the start of a dawn song as the time at which a bird sang its first song and the end as the time when the bird stopped singing for longer than 7 min [69,70]. We determine that the repertoire size followed existing song-type categorization criteria for great tits [71]. All measurements were collected by the same person. We could not consider within-individual variation during the same season; however, repertoire size was not found to differ between breeding stages [69].

### 2.6. Statistical Analyses

A double-breeding pair was defined as one having a second clutch following a successful first clutch (at least one fledgling leaving the nest) in the same breeding season regardless of whether the second brood was successful [71]. A single-breeding pair was defined as one having only one clutch in the given breeding season.

To examine the effect of the date of the first egg of the first brood and of female quality on double breeding, we used generalized linear mixed models (GLMMs) with a logit-link and binomial error distribution. In the model of the date of the first egg of the first brood, the response variable was whether the pair initiated a second brood (1 and 0, respectively). The explanatory variable was the first brood-laying date. We included female identity (ID) and year as random effects.

In the model of the effect of female quality on double breeding, the response variable was whether the pair initiated a second brood (1 and 0, respectively). The explanatory variables were the tarsus length, individual heterozygosity (Hs), and individual *F*. We included year and female identity (ID) as random effects. Multicollinearity of the independent variables was tested prior to analysis using the variance inflation factor (VIF) and was not detected (i.e., VIF < 4).

To examine the effect of the number of offspring of the first clutch on the pairing status of male great tits in double breeding, we used generalized linear mixed models (GLMMs) with a logit-link and binomial error distribution. The response variable was whether the pair initiates a second brood (1 and 0, respectively). The explanatory variable was the number of offspring of the first clutch. We included male identity (ID) and year as random effects.

To examine the patterns that female great tits use for social mate choice in double breeding, we built three generalized linear mixed models (GLMMs) considering three hypotheses: (a) selection according to good genes (tarsus length, breast stripe width, repertoire size, individual heterozygosity (Hs), and individual *F*), (b) selection according to compatible genes (relatedness), or (c) selection according to both good genes and compatible genes. The GLMMs involved a logit link function and binomial error distribution. The dependent variable was whether the male was involved in double breeding (1 and 0, respectively). We included male identity (ID) as a random effect to account for pseudo-replication, as some individuals bred in multiple years at our site. We also included year as a random effect to account for differences in the incidence of multiple brooding across the years of study. The independent variables were (a) tarsus length, breast black stripe width, repertoire size, individual *F*, and heterozygosity (Hs) of males; (b) relatedness (*r*) of pairs; and (c) tarsus length, breast black stripe width, repertoire size, individual *F*, Hs, and *r* of pairs. Only successful nests (nests in which at least one nestling fledged) were included in the analysis. Then, we compared the Akaike Information Criterion (AIC) values to evaluate the relative importance of the models [20,72].

We also examined female preference in choosing males with particular traits as social mates for double breeding. First, we addressed the main characteristics influencing mate choice by female great tits in double breeding by testing the effect of each variable with model selection. For this analysis, a global model including all explanatory variables was constructed. We then fitted all possible nested models, ranked them according their AICc (small sample size-adjusted Akaike’s information criterion) value [73], then selected the models with ΔAICc < 4, and calculated predictions. Multicollinearity of the independent variables was tested prior to analysis using the variance inflation factor (VIF) and was not detected (i.e., VIF < 2).

Next, we examined whether double-breeding females tend to mate with genetically dissimilar males to form social bonds using randomization tests of relatedness. To test this hypothesis, we calculated the distribution of average relatedness under the null hypothesis: double-breeding females choose socially males irrespective of their relatedness. The computation allowed us to determine the average relatedness between those dyads (all other male–female pair combinations possible in each year) [10]. We iterated the computation 10,000 times and obtained a distribution of average relatedness. We obtained two critical values at the 2.5% ends of this distribution. When the observed average is out of the range of the two critical values, the null hypothesis is rejected [18].

To evaluate whether double-breeding females prefer males with other characteristics as social mates, for example, on the basis of stripe size, the null hypothesis was that double-breeding females choose socially males irrespective of their breast stripe size. We calculated the average of the trait values of double-breeding males as the expected value and calculated critical values for the average trait value of single-breeding males based on 10,000 random values as described above. Similar randomization tests were performed for individual heterozygosity (Hs).

When assessing whether double-breeding males tend to have “better” characteristics (larger stripe size, for example) than the single-breeding males, the differences between double-breeding males and single-breeding males were examined using independent-sample *t*-tests or Wilcoxon signed-rank tests.

To evaluate whether the gene quality differed between the offspring of double- and single-breeding pairs, we compared the individual heterozygosity and individual *F* values between offspring of double- and single-breeding pairs using the Wilcoxon signed-rank test.

All statistical analyses were performed in R 3.3.2 (R Development Core Team, http://cran.r-project.org/) with the packages “*lme4*” for GLMM construction and “*MuMIn*” for model selection.

## 3. Results

Seventy-three clutches were captured for which both adults were known (29 in 2014, 23 in 2015, and 21 in 2016). Overall, 22 pairs (30.1%) initiated second breeding attempts, mating again with the same mate. The number of offspring of double-breeding pairs (mean ± SE: 19.77 ± 0.431) is significantly more than single-breeding pairs (mean ± SE: 11.80 ± 0.208; *W*_22,51_ = 1122, *p* < 0.001). Male dawn songs were recorded for 63 nests. The characteristic values (mean ± SE) of males are shown in Table 1.

### 3.1. The Timing of Breeding and Female Quality

**Figure 1:**
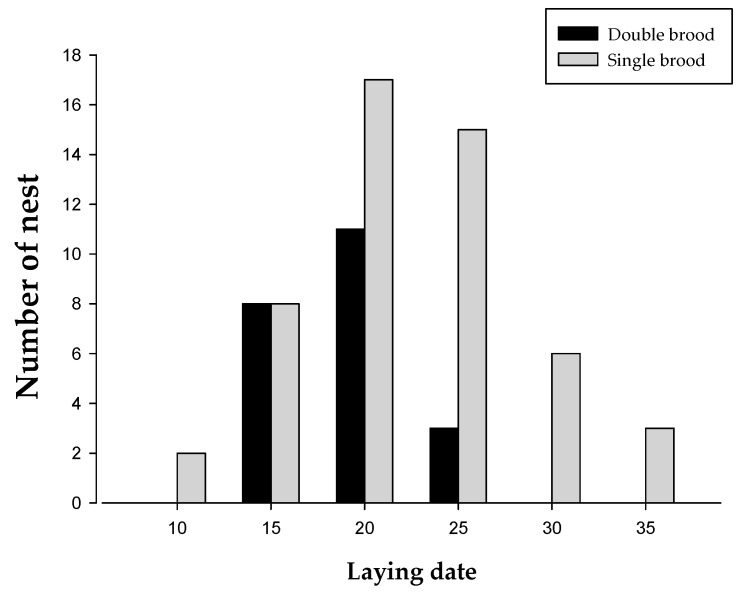
Frequency distribution of laying date (1 = 1 Apr) of the first broods (*n* = 22) and single broods (*n* = 51) in the great tits.

The incidence of second clutches is not affected by the first egg-laying date (GLMM: χ^2^ = 1.712, *p* = 0.191). Nevertheless, the first brood-laying date of double brooding was early and concentrated in the first half period (the dataset comprised the laying date of the first brood of double and single broods) (Figure 1), the first egg-laying date of double breeder was April 17 (mean ± SE = 17.182 ± 0.692, *n* = 22), and that of the single breeder was April 20 (mean ± SE = 20.451 ± 0.822, *n* = 51).

Female quality is not the main factor influencing breeder initiation of second clutches (Table 2). However, the individual heterozygosity of double-breeding females is higher than single-breeding females but does not reach a significant level (*p* = 0.054).

### 3.2. Good Genes, Compatible Genes, or Both

The results from the evaluation of double-breeding great tit female choice based on genetic benefit signals are shown in Table 3. The model combining both good genes and compatible genes, which included six variables (tarsus length, black stripe width, repertoire size, individual *F* and heterozygosity, and relatedness), showed the minimum AIC.

### 3.3. Female Trait Preference in Choosing Males for Double Breeding

The ranking of the 64 candidate models for predicting pairing status of male great tits in double breeding showed 5 models with ΔAICc < 4 (Table 4). Individual *F* and tarsus length were absent from most top models, and neither parameter was significantly associated with males paired with double breeding (Table 5). The repertoire size was not an important factor associated with males paired with double breeding, as it was absent from all top models (Table 4). The relatedness of pairs and breast black stripe width were included in all top models, and heterozygosity was present in all but one of the top models (Table 3), indicating that these variables are the most important factors associated with male pair status in double breeding (Table 5).

**Figure 2:**
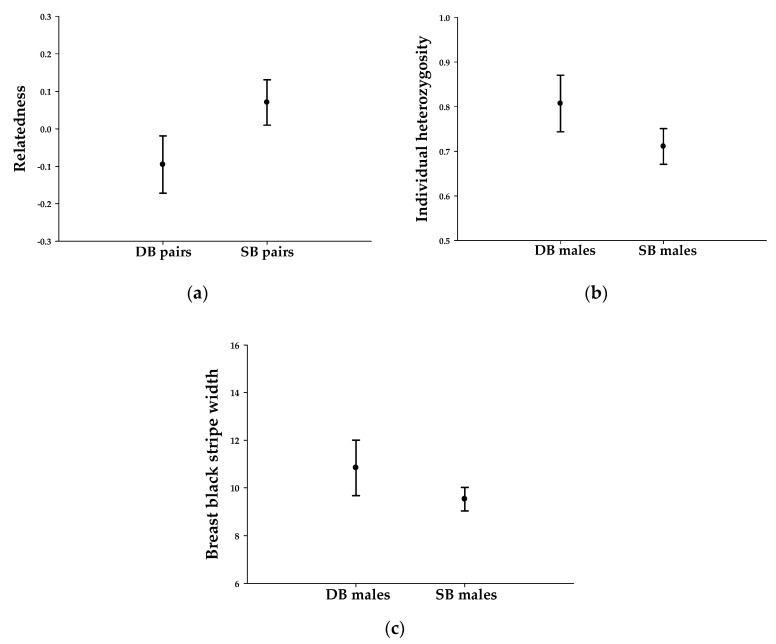
(**a**) Relatedness of double-breeding (DB) and single-breeding (SB) pairs; (**b**) individual heterozygosity for double-breeding (DB) and single-breeding (SB) males; and (**c**) breast black stripe width for double-breeding (DB) and single-breeding (SB) males: the results are presented as the means and upper–lower 95% confidence intervals for each group.

We examined whether double-breeding females tend to mate with genetically dissimilar males to form social bonds with high heterozygosity and a large black stripe on the breast. The observed average relatedness between a double-breeding female and her social partner deviated from the expectation under the null hypothesis: females engaged in double breeding more often with a male with low relatedness than with a random male from the population (the critical values were −0.062 and −0.056, respectively, and the average relatedness of the 22 observed pairs was −0.096). In addition, double-breeding pairs generally showed lower relatedness than single-breeding pairs (*t*_22,51_ = 3.489, *p* = 0.001, Figure 2a). The observed average heterozygosity of double-breeding males deviated from the expectation under the null hypothesis: males with higher heterozygosity were more likely to be paired with double breeding than those with lower heterozygosity (the critical values were 0.708 and 0.714, respectively, and the average among the observed males was 0.807). Furthermore, double-breeding males showed higher heterozygosity than single-breeding males (*W*_22,51_ = 374, *p* = 0.020, Figure 2b). The observed average stripe size of double-breeding males deviated from the expectation under the null hypothesis: males with a large breast black stripe were more likely be paired to double breeding than those with a smaller stripe (the critical values were 9.490 and 9.559, respectively, and the average among observed males was 10.838). In addition, double-breeding males had a larger breast black stripe than single-breeding males (*t*_22,51_ = 2.144, *p* = 0.040, Figure 2c).

### 3.4. Genetic Quality and Number of Offspring

The number of nestlings from the first clutch is not the most important factors associated with male pair status in double breeding (GLMM: χ^2^ = 0.743, *p* = 0.389, first broods: mean ± SE = 11.591 ± 0.234, *n* = 22; single broods: mean ± SE = 11.804 ± 0.208, *n* = 51).

**Figure 3:**
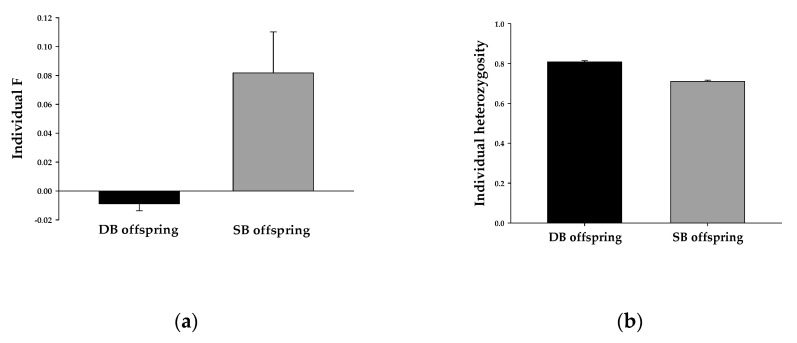
(**a**) Individual F of double-breeding (DB) and single-breeding (SB) offspring, and (**b**) individual heterozygosity of double-breeding (DB) and single-breeding (SB) offspring: the results are presented as the means and SE for each group.

We focused on the variation in individual *F* and individual heterozygosity among offspring and compared the individual *F* and individual heterozygosity of the offspring of double-breeding pairs and single-breeding pairs. The offspring of double-breeding pairs had a lower individual *F* (double-breeding offspring (mean ± SE): −0.009 ± 0.005, single-breeding offspring (mean ± SE): 0.082 ± 0.028, *W*_416,565_ = 75,723, *p* < 0.001, Figure 3a) and higher individual heterozygosity (double-breeding offspring (mean ± SE): 0.808 ± 0.007, single-breeding offspring (mean ± SE): 0.710 ± 0.006, *W*_416,565_ = 161,468, *p* < 0.001, Figure 3b) than those of single-breeding pairs.

## 4. Discussion

In the present study, the date of the first egg of the first brood and female quality do not affect initiation of the second brood but the individual heterozygosity of double-breeding females are slightly higher than that of single-breeding females. We investigated the method of female choice based on genetic benefits in double-breeding great tits. Female great tits choose males with both compatible genes and good genes for double-breeding mating. Double-breeding females prefer males with large breast stripes, high heterozygosity, and low relatedness. The number of offspring of the first clutch does not affect the pairing status of males for double breeding. The genetic quality of offspring of double-breeding pairs may be higher than that of single-breeding pairs (higher heterozygosity and lower individual *F*).

Theoretically, double breeders will commence first broods as early as possible to be able to produce subsequent clutches [74] and earlier breeders are likely to have better individual quality, which is beneficial for initiated second clutches. However, in our research area, the date of first egg of double breeders is not significantly earlier than single breeders. Initiated clutches that are too early could result in high costs due to low temperatures [75], affecting the energy input of subsequent breeding and the length of a breeding cycle. Temperature affects the timing of peaks in spring caterpillar abundance [76]. At cold temperatures, parents have difficulty finding food for themselves and their offspring [77]; a large amount of time is spent foraging, which results in extended incubation [78] and nestling periods [79]; and offspring will also be directly injured by the low temperature. High-quality breeders generally breed relatively earlier, and too late of an initiation may result in the remaining effective breeding time not being sufficient to breed two clutches. Therefore, the date of the first egg of double breeders is not significantly earlier (concentrated in the first half period).

In our study area, individual heterozygosity slightly influenced the female to initiate a second breeding. In various species, heterozygosity influences individual fitness. High individual heterozygosity could increase the number of potentially useful genes or reduce the likelihood of recessive deleterious allele expression. Individuals with high heterozygosity generally have higher reproductive performance [80], resistance to parasites [81], and survival [82]. Female conditions are known to affect the propensity of double breeding; however, our results do not reach a significant level. This may due, in addition to genetic quality, to reproductive experience (age), which may have a greater impact on double breeding [38]. However, we did not collect data on female age. It may also be a question of insufficient sample size.

The offspring of the first clutch may affect the activity and investments of the adults and their willingness for a second brood. In theory, the probability of double brooding usually declines with increasing offspring of the first clutch due to the limited amount of energy that can be allocated to first and second clutches [35,41]. However, females that mated with higher-quality males may increase their reproductive effort by producing more offspring [83,84]. The males who are paired to double breeding females general have higher individual quality. Our results show that the number of offspring of the first clutch does not affect the pairing status of male great tits in double breeding, which may be due to the trade-off of the breeder. Our results show that the number of offspring of the first clutch does not affect the pairing status of male great tits in double breeding.

Genetic quality mainly has two components: good genes and compatible genes. Females mate with males who will contribute good or compatible genes to their offspring, thereby increasing the genetic quality of their offspring. “Good males” could pass on good genes to their offspring and could produce highly fit offspring regardless of the contribution of maternal genetic. While compatible genes are dependent on the interaction of pairs and genotypes, compatible males should produce high-quality offspring when matched with a specific maternal haplotype; therefore, pairings are needed to increase fitness of offspring. There is a potential trade-off in mate choice between good genes and compatible genes [85]. Females may adjust their mate choice method according to the prevailing conditions at any particular time, and they can preferentially choose compatible genes or good genes. When there is a certain equilibrium between the good gene and compatible gene fitness effects, the two mating systems may coexist. They are not mutually exclusive, and the combined effect of “good genes” and compatibility in mate choice preference may be optimized by individuals at the genetic level. Female great tits chose males with both compatible genes and good genes in double-breeding mating. The genetic quality of the offspring of double-breeding pairs was higher than that of the offspring of single-breeding pairs (higher heterozygosity and lower individual *F*). This, to some extent, validates the advantages of the mate choice model. Producing two broods within the same season is energy-consuming and may affect the future survival of the parents, which requires individuals to have better quality to bear the cost. Therefore, females will use this more complex mating decisions to choose better males.

Most mate choice theories suggest that females prefer high-quality compared to low-quality males [86]. Phenotypic traits can reflect the intrinsic quality of individuals. Females may use these traits to choose males who with “good genes”, thereby increasing their fitness. Breast stripe size is an indicator of male genetic quality and is repeatable and heritable. This is thought to be related to dominance [56,57], nest defensive [17,59], and nest attentiveness [17]. Therefore, double-breeding females may benefit from choosing males with a large breast stripe. Female great tits paired with males with a large breast stripe lay large clutches, and their offspring showed greater viability or a heavier body weight than those of females paired with males with a smaller stripe [14]. Therefore, double-breeding females may benefit from choosing males with a large breast stripe.

Males with high heterozygosity have a distinct advantage. Heterozygosity is positively correlated with fitness, especially in species with biparental care; it has direct effects on reproductive success. However, a negative effect of individual heterozygosity on reproductive success was found in blue tits [87]. This may be due to methodological limitations related to the sample size and/or the number of markers used [88], the study timespan [89], the life-history stages considered [90], or the type and number of traits assessed [28]. Overall, most studies suggest that individual heterozygosity has a positive impact on fitness. Heterozygous individuals have been shown to lay larger clutches [9], to have better territories [91,92], and to feed their offspring more often [5] than non-heterozygous individuals. Furthermore, in addition to active female mate selection, mechanisms such as male–male competition may have an impact on double-breeding mate choice patterns. Males with high heterozygosity may have more advantages in intrasexual competition and thus have more opportunities to be selected by females. Mating with heterozygous males, females may gain indirect benefits while also gaining direct benefits, which is conducive to double breeding.

High genetic similarity of mates has long been known to reduce the reproductive success of pairs and fitness of offspring [32]. The genetic similarity of social mates not only can predict hatching success but also may affect offspring fitness of later life stages, including during the nestling period and beyond. As the genetic similarity of pairs increases, the weight and growth rate of offspring [93], fledging success [34], and offspring immunocompetence [94] significantly decline. Therefore, double-breeding females prefer to choose genetically dissimilar males.

In the current study, female preference for a large repertoire size in male song was not evident. Singing is a time-consuming and energy-consuming activity, and males may exhibit a tradeoff between parental investment and continued display. Furthermore, as built into the handicap models, the energetic costs of singing will impose direct costs onto those expressing it [95], and excessive energy consumption is not conducive to initiated second clutches. A preference among females in terms of tarsus length or individual *F* was also not observed in this study.

Mating with heterozygous and compatible males should increase offspring heterozygosity. Heterozygous offspring may be healthier than non-heterozygous offspring [96] and may display attractive secondary sexual traits [9]. They may also tend to share more alleles with the average potential mates and may thus have more advantages in mate choice. Males with a large breast stripe and high heterozygosity may not only pass on good genes to their offspring but also may have access to more high-quality resources or may exhibit greater parental investment than those with a smaller stripe and lower heterozygosity. The benefits of producing two broods within the same season are obvious, but so are the costs. Choosing a high-quality male not only may improve the genetic quality of the offspring but also may facilitate the successful completion of double breeding. In previous studies, adaptive female choice varied among generations or among populations [85] or changed temporally on the basis of ornamentation and relatedness among available mates [97].

## 5. Conclusions

This study confirms that double-breeding female great tits adopt multiple patterns for genetic benefits to choose mates. We first analyzed the effect of the first egg-laying date and female quality on double breeding in a Northeast China population of great tits and found that there was a trade-off between the benefits and cost, that the date of the first egg of double breeders is not significantly earlier (concentrated in the first half period), and that female individual heterozygosity is slightly influenced to initiate a second breeding. We tested two hypotheses regarding double-breeding female choice and found that female great tits choose males with both compatible genes and good genes in double-breeding mating. Double-breeding females prefer males with large breast stripes, high heterozygosity, and low relatedness. The number of offspring of the first clutch did not affect the pairing status of male great tits in double breeding. The offspring of double-breeding pairs have higher heterozygosity and lower individual *F* than that of single-breeding pairs. In this selection mode, double-breeding pairs simultaneously improve the quality and the number of offspring, thereby maximizing reproductive fitness. Uncovering the patterns used in mate choice for genetic benefits adopted by double-breeding females would provide a better understanding of the life history and rules of female choice.

## Figures and Tables

**Table 1:** Basic statistics (mean ± S.E. and range) for the different female and male characteristics analyzed in the present study.

Male Characteristics	Single Breeding		Double Breeding	
	Female	Male	Female	Male
Individual *F*	0.012 ± 0.009 (*n* = 51)	0.037 ± 0.017 (*n* = 51)	0.002 ± 0.018 (*n* = 22)	0.004 ± 0.024 (*n* = 22)
Individual heterozygosity (Hs)	0.725 ± 0.018 (*n* = 51)	0.712 ± 0.020 (*n* = 51)	0.790 ± 0.039 (*n* = 22)	0.807 ± 0.030 (*n* = 22)
Breast black stripe width	-	9.525 ± 0.256 (*n* = 51)	-	10.838 ± 0.561 (*n* = 22)
Tarsus length	21.298 ± 0.139 (*n* = 51)	21.690 ± 0.188 (*n* = 51)	21.553 ± 0.262 (*n* = 22)	21.216 ± 0.264 (*n* = 22)
Repertoire size	-	3.55 ± 0.174 (*n* = 42)	-	3.95 ± 0.381 (*n* = 21)
Relatedness	-	0.070 ± 0.030 (*n* = 51)	-	−0.096 ± 0.037 (*n* = 22)

**Table 2:** Results of the generalized linear mixed models (GLMMs) of female-quality influence on the probability of double brooding.

Variable	Estimate	se	df	*Z*	*p*
Intercept	−12.829	6.948	1	−1.846	0.065
Individual *F*	8.995	8.109	1	1.265	0.206
Individual heterozygosity (Hs)	7.019	3.650	1	1.923	0.054
Tarsus length	0.303	0.268	1	1.131	0.258

**Table 3:** The AIC of each GLMM predicting the pairing status of male great tits in double breeding.

	Model	AIC
Good	Individual *F* + Individual heterozygosity + Breast black stripe width + Tarsus length + Repertoire size	84.7
Compatible	Relatedness	87.2
Good and Compatible	Individual *F* + Individual heterozygosity + Breast black stripe width + Tarsus length + Repertoire size + Relatedness	76.4

**Table 4:** Selection of the GLMMs for predicting pairing status of male great tits in double breeding in relation to individual *F*, individual heterozygosity, breast black stripe width, tarsus length, and repertoire size: the number of estimated parameters (np), model deviance, difference in AICc between the current and the best model (ΔAICc), and AICc weights (ω_i_) are given for each model.

NO.	Model	np	Δ AIC_C_	Deviance	ω_i_
1	Individual heterozygosity + Relatedness+ Breast black stripe width	6	0.00	65.8	0.34
2	Tarsus length + Individual heterozygosity + Relatedness + Breast black stripe width	7	0.98	64.3	0.21
3	Individual heterozygosity + Relatedness + Breast black stripe width + Individual *F*	7	2.41	65.8	0.10
4	Tarsus length + Heterozygosity + Relatedness + Breast black stripe width + Individual *F*	8	3.33	64.2	0.07
5	Tarsus length + Relatedness + Breast black stripe width	6	3.75	66.4	0.05

**Table 5:** Parameter estimates and *p* values of the fitted top GLMMs (ΔAICc < 4).

Variable	Estimate	se	df	*Z*	*p*
Intercept	−6.347	5.860	1	1.072	0.284
Individual *F*	−0.891	2.872	1	0.305	0.761
Individual heterozygosity (Hs)	5.866	2.653	1	2.172	0.030
Breast black stripe width	0.384	0.161	1	2.343	0.019
Tarsus length	−0.295	0.234	1	1.239	0.215
Relatedness	−5.124	1.842	1	2.731	0.006

## Data Availability

The data presented in this study are available on request from the corresponding author. The data are not publicly available due to part of the data will also be used for unpublished articles.

## Appendix A

**Table A1:** Panel of 14 microsatellite markers used in the present study: a subset of 8 markers (denoted with an asterisk) was selected for parentage analyses.

Locus	Sequence	Repeat Type	Expected Allele Size (bp)	Annealing Temperature
Pma33	F-TTCCCCAAGTATCCTGCATC R-AAACCATATCACCCAGTGCC	(GATA)_14_GAT(GATA)_8_	305	55
Pma1 *	F-CTCTTTTCCCAGCCTCCAG R-TATGTTTTGTCTGCTCGGGG	(CA)_15_(CT)(CA)_4_	118	58
Pma30 *	F-GTTTCTGCCCAAATGGTGTG R-TCAGACCTTTCCAATGATGG	(GA)_10_	305	58
Pma179 *	F-GGAGGCTTAAACATTCTGTGTG R-GGGCTGAAGGAGTTTGCTAC	(TG)_14_	179~197	61
Pmad22	F-GATCAGAGCTTGCCTCAACAC R-TCTGGGCTGAAATACCTACCC	(CTAT)_15_(CCAT)_12_	403	60
Pma42 *	F-ACTTCCACATGCCAGTTTCC R-TGTTAAGGCAGAGAGGTGGG	(TCCA)_15_	285	57
Pma27 *	F-TATAAACCACAGCCACACGC R-CACAACCACAGAGGCATGAG	(CAT)_16_	202	55
Pma40	F-CGTTCCTCCTTTGCTTTCTG R-AATGGCACAACACCTTCTCC	(GA)_10_	416	58
Pma71 *	F-TCAGCCTCCAAGGAAAACAG R-GCATAAGCAACACCATGCAG	(TAGG)_6_(TAGA)_11_	186	58
Pma45 *	F-CCCCTGGCTCTTTCATATCC R-GACAGGTGTTGGCACAAGG	(TGA)_10_	307	58
PmaC25 *	F-CGTCCTGCTGTTTGTATTTCTG R- CCATGAACCATTTTTAGGGTG	(CAT)_11_	323	58
PmaD105	F- CAAATCACACAGTTGCTGCC R-CCTGGTATAAGACTGGTCAAAACAG	(GTCT)_3_(ATCT)_12_	404	58
PmaGAn28	F- GTTGGTGCAGCGGTCTACTC R-CATGTTGGGACAGCAGTTTG	(GA)_16_	199	58
Pma48m	F- CACTCAGCCTCTCAGATCTG R-CGGGCTGGTACTTATTGGGAG	(TG)_11_	192	58

## References

[1] Kirkpatrick M., Barton N.H. (1997). The strength of indirect selection on female mating preferences. Proc. Natl. Acad. Sci. USA.

[2] Jennions M.D., Petrie M. (2007). Why do females mate multiply? A review of the genetic benefits. Biol. Rev..

[3] Consuegra S., De Leaniz C.G. (2008). MHC-mediated mate choice increases parasite resistance in salmon. Proc. R. Soc. B Biol. Sci..

[4] Neff B.D., Pitcher T.E. (2004). Genetic quality and sexual selection: An integrated framework for good genes and compatible genes. Mol. Ecol..

[5] Tregenza T., Wedell N. (2000). Genetic compatibility, mate choice and patterns of parentage: Invited Review. Mol. Ecol..

[6] Mays H.L., Hill G.E. (2004). Choosing mates: Good genes versus genes that are a good fit. Trends Ecol. Evol..

[7] Kempenaers B., Verheyen G.R., Van Broeckhoven C., Burke T., Van Broeckhoven C., Dhondt A. (1992). Extra-pair paternity results from female preference for high-quality males in the blue tit. Nat. Cell Biol..

[8] Hasselquist D., Bensch S., Von Schantz T. (1996). Correlation between male song repertoire, extra-pair paternity and offspring survival in the great reed warbler. Nat. Cell Biol..

[9] Foerster K., Delhey K., Johnsen A., Lifjeld J.T., Kempenaers B. (2003). Females increase offspring heterozygosity and fitness through extra-pair matings. Nat. Cell Biol..

[10] Wright D.J., Brouwer L., Mannarelli M.-E., Burke T., Komdeur J., Richardson D.S. (2015). Social pairing of Seychelles warblers under reduced constraints: MHC, neutral heterozygosity, and age. Behav. Ecol..

[11] Rowe L., Houle D. (1996). The lek paradox and the capture of genetic variance by condition dependent traits. Proc. R. Soc. B Biol. Sci..

[12] Trivers R.L., Campbell B. (1972). Parental investment and sexual selection. Sexual Selection and the Descent of Man.

[13] Drickamer L.C. (1992). Oestrous female house mice discriminate dominant from subordinate males and sons of dominant from sons of subordinate males by odour cues. Anim. Behav..

[14] Norris K. (1993). Heritable variation in a plumage indicator of viability in male great tits Parus major. Nat. Cell Biol..

[15] Hamilton W.D., Zuk M. (1982). Heritable true fitness and bright birds: A role for parasites?. Science.

[16] Kawano K.M., Yamaguchi N., Kasuya E., Yahara T. (2008). Extra-pair mate choice in the female great tit Parus major: Good males or compatible males. J. Ethol..

[17] Norris K.J. (1990). Female choice and the quality of parental care in the great tit Parus major. Behav. Ecol. Sociobiol..

[18] Senar J.C., Quesada J. (2009). Cross-fostering experiments to compare carotenoid- and melanin-based plumage traits and long-term parental effects in post-moulted great tits. Behaviour.

[19] Senar J.C., Conroy M.J., Quesada J., Mateos-Gonzalez F. (2014). Selection based on the size of the black tie of the great tit may be reversed in urban habitats. Ecol. Evol..

[20] Byers B.E., Kroodsma D.E. (2009). Female mate choice and songbird song repertoires. Anim. Behav..

[21] Sung H.-C., Handford P.T. (2020). Song characters as reliable indicators of male reproductive quality in the Savannah Sparrow (Passerculus sandwichensis). Can. J. Zool..

[22] Krebs J.R., Kacelnik A. (1983). The Dawn Chorus in the great tit (Parus Major): Proximate and Ultimate Causes. Behaviour.

[23] Slagsvold T., Sætre G.-P., Dale S. (1994). Dawn singing in the Great Tit (Parus Major): Mate attraction, mate guarding, or territorial defence?. Behaviour.

[24] Brown J.L. (1997). A theory of mate choice based on heterozygosity. Behav. Ecol..

[25] Mitton J.B., Schuster W.S.F., Cothran E.G., De Fries J.C. (1993). Correlation between the individual heterozygosity of parents and their offspring. Heredity.

[26] Hoffman J.I., Forcada J., Trathan P.N., Amos W. (2007). Female fur seals show active choice for males that are heterozygous and unrelated. Nat. Cell Biol..

[27] Kempenaers B. (2007). Mate choice and genetic quality: A review of the heterozygosity theory. Adv. Study Behav..

[28] Chapman J.R., Akagawa S.N., Oltman D.W.C., Late J.S., Heldon B.C.S. (2009). A quantitative review of heterozygosity-fitness correlations in animal populations. Mol. Ecol..

[29] García-Navas V., Ortego J., Sanz J.J. (2009). Heterozygosity-based assortative mating in blue tits (Cyanistes caeruleus): Implications for the evolution of mate choice. Proc. R. Soc. B Biol. Sci..

[30] Zeh J.A., Zeh D.W. (1996). The Evolution of Polyandry I: Intragenomic Conflict and Genetic Incompatibility.

[31] Charlesworth A.D. (1987). Inbreeding depression and its evolutionary consequences. Ann. Rev. Ecol. Syst..

[32] Amos W., Wilmer J.W., Fullard K., Burg T.M., Croxall J.P., Bloch D., Coulson T. (2001). The influence of parental relatedness on reproductive success. Proc. R. Soc. B Biol. Sci..

[33] Szulkin M., Zelazowski P., Nicholson G., Sheldon B.C. (2009). Inbreeding avoidance under different null models of random mating in the great tit. J. Anim. Ecol..

[34] Van De Casteele T., Galbusera P., Schenck T., Matthysen E. (2003). Seasonal and lifetime reproductive consequences of inbreeding in the great tit Parus major. Behav. Ecol..

[35] Nagy L.R., Holmes R.T. (2005). To double-brood or not? Individual variation in the reproductive effort in black-throated blue warblers (Dendroica caerulescens). Auk.

[36] O’Brien E.L., Dawson R.D. (2012). Experimental dissociation of individual quality, food and timing of breeding effects on double-brooding in a migratory songbird. Oecologia.

[37] Verhulst S., Tinbergen J.M., Daan S. (1997). Multiple breeding in the Great Tit. A trade-off between successive reproductive attempts?. Funct. Ecol..

[38] Hoffmann J., Postma E., Schaub M. (2014). Factors influencing double brooding in Eurasian Hoopoes Upupa epops. Ibis.

[39] Winkel W., Winkel D. (1995). Kosten und Nutzen von Zweitbruten bei der Tannenmeise (Parus ater) Costs and benefits of second broods in Coal Tits (Parus ater). J. Ornithol..

[40] Harrison T.J.E., Smith J.A., Martin G.R., Chamberlain D.E., Bearhop S., Robb G.N., Reynolds S.J. (2010). Does food supplementation really enhance productivity of breeding birds?. Oecologia.

[41] Verboven N., Verhulst S. (1996). Seasonal variation in the incidence of double broods: The date hypothesis fits better than the quality hypothesis. J. Anim. Ecol..

[42] Harvey P.H., Greenwood P.J., Perrins C.M., Martin A.R. (1979). Breeding success of great tits Parus Major in relation to age of male and female parent. Ibis.

[43] Jankowiak Ł., Wysocki D. (2015). Do individual breeding experience and parental effort affect breeding season length in blackbirds?. Behav. Ecol..

[44] Whelan S., Strickland D., Morand-Ferron J., Norris D.R. (2016). Male experience buffers female laying date plasticity in a winter-breeding, food-storing passerine. Anim. Behav..

[45] Husby A., Kruuk L.E., Visser M.E. (2009). Decline in the Frequency and Benefits of Multiple Brooding in Great Tits as a Consequence of a Changing Environment.

[46] Wolf L., Ketterson E.D., Nolan V. (1991). Female condition and delayed benefits to males that provide parental care: A removal study. Auk.

[47] Monroe A.P., Hallinger K.K., Brasso R.L., Cristol D.A. (2008). Occurrence and implications of double brooding in a southern population of tree swallows. Condor.

[48] Nilsson J.Å., Smith H.G. (1988). Incubation feeding as a male tactic for early hatching. Anim. Behav..

[49] Cantarero A., López-Arrabé J., Palma A., Redondo A.J., Moreno J. (2014). Males respond to female begging signals of need: A handicapping experiment in the pied flycatcher, Ficedula hypoleuca. Anim. Behav..

[50] Verhulst S., Hut R.A. (1996). Post-fledging care, multiple breeding and the costs of reproduction in the great tit. Anim. Behav..

[51] Del Hoyo J., del Hoyo J., Elliott A., Christie D.A. (2007). Handbook of the Birds of the World Vol. 12 Picathartes to Tits and Chickadees.

[52] Nomi D., Yuta T., Koizumi I. (2017). Male feeding contribution facilitates multiple brooding in a biparental songbird. Ibis.

[53] Sun H., Gao W., Gong L., Yang Y.L., Wang H.T. (2008). The study of composition and diversity of birds in Zuojia nature preserved area, Jilin Province. J. Northeast. Norm. Univ. (Nat. Sci. Ed.).

[54] Senar J.C., Pascual J. (1997). Keel and tarsus length may provide a good predictor of avian body size. Ardea.

[55] Lemel J., Wallin K. (1993). Status signalling, motivational condition and dominance: An experimental study in the great tit, *Parus major* L.. Anim. Behav..

[56] Järvi T., Bakken M. (1984). The function of the variation in the breast stripe of the great tit (Parus major). Anim. Behav..

[57] Poeysae H. (1988). Feeding consequences of the dominance status in great tit Parus major groups. Ornis Fenn..

[58] Figuerola J., Senar J.C. (2008). Measurement of plumage badges: An evaluation of methods used in the Great Tit Parus major. Ibis.

[59] Quesada J., Senar J.C. (2007). The role of melanin- and carotenoid-based plumage coloration in nest defence in the Great Tit. Ethology.

[60] Harper D.G. (1994). Some comments on the repeatability of measurements. Ringing Migr..

[61] Saladin V., Bonfils D., Binz T., Richner H. (2003). Isolation and characterization of 16 microsatellite loci in the Great Tit Parus major. Mol. Ecol. Notes.

[62] Kawano K.M. (2003). Isolation of polymorphic microsatellite markers in the great tit (Parus major minor). Mol. Ecol. Notes.

[63] Marshall T.C., Slate J., Kruuk L.E.B., Pemberton J.M. (1998). Statistical confidence for likelihood-based paternity inference in natural populations. Mol. Ecol..

[64] Goudet J. (1995). FSTAT (Version 1.2): A computer program to calculate F-statistics. J. Hered..

[65] Dawson D.A., Horsburgh G.J., Küpper C., Stewart I.R.K., Ball A.D., Durrant K.L., Hansson B., Bacon I., Bird S., Klein Á. (2010). New methods to identify conserved microsatellite loci and develop primer sets of high cross-species utility–as demonstrated for birds. Mol. Ecol. Resour..

[66] Gompert Z. (2011). Coancestry: A program for simulating, estimating and analysing relatedness and inbreeding coefficients. Mol. Ecol. Resour..

[67] Ritland K. (1996). Estimators for pairwise relatedness and individual inbreeding coefficients. Genet. Res..

[68] García-Navas V., Ferrer E.S., Cáliz-Campal C., Bueno-Enciso J., Barrientos R., Sanz J.J., Ortego J. (2015). Spatiotemporal and genetic contingency of extrapair behaviour in a songbird. Anim. Behav..

[69] Naguib M., Diehl J., Van Oers K., Snijders L. (2019). Repeatability of signalling traits in the avian dawn chorus. Front. Zool..

[70] Bircher N., Van Oers K., Hinde C.A., Naguib M. (2020). Extraterritorial forays by great tits are associated with dawn song in unexpected ways. Behav. Ecol..

[71] McGregor P.K., Krebs J.R., Perrins C.M. (1981). Song repertoires and lifetime reproductive success in the great tit (Parus major). Am. Nat..

[72] Crawley M.J. (2002). Statistical Computing: An Introduction to Data Analysis Using S-Plus.

[73] Jørgensen S. (2004). Model selection and multimodel inference. Ecol. Model..

[74] Crick H.Q.P., Gibbons D.W., Magrath R.D. (1993). Seasonal changes in clutch size in British birds. J. Anim. Ecol..

[75] Visser M., Van Noordwijk A.J., Tinbergen J.M., Lessells C.M. (1998). Warmer springs lead to mistimed reproduction in great tits (Parus major). Proc. R. Soc. B Biol. Sci..

[76] Thorley J.B., Lord A.M. (2015). Laying date is a plastic and repeatable trait in a population of blue tits Cyanistes caeruleus. Ardea.

[77] Virkkala R. (1990). Ecology of the siberian Tit Parus cinctus in relation to habitat euality: Effects of forest management. Ornis Scand..

[78] White F.N., Kinney J.L. (1974). Avian incubation. Science.

[79] Stodola K.W., Buehler D.A., Kim D.H., Franzreb K.E., Linder E.T. (2010). Biotic and abiotic factors governing nestling-period length in the ovenbird (Seiurus Aurocapilla). Auk.

[80] Ortego J., Calabuig G., Bonal R., Muñoz A., Aparicio J.M., Cordero P.J. (2009). Temporal variation of heterozygosity-based assortative mating and related benefits in a lesser kestrel population. J. Evol. Biol..

[81] Acevedo-Whitehouse K., Spraker T.R., Lyons E., Melin S.R., Gulland F., Delong R.L., Amos W. (2006). Contrasting effects of heterozygosity on survival and hookworm resistance in California sea lion pups. Mol. Ecol..

[82] Olano-Marin J., Mueller J.C., Kempenaers B. (2011). Heterozygosity and survival in blue tits (Cyanistes caeruleus): Contrasting effects of presumably functional and neutral loci. Mol. Ecol..

[83] Gil D., Graves J., Hazon N., Wells A. (1999). Male attractiveness and differential testosterone investment in Zebra Finch eggs. Science.

[84] Petrie M., Williams A. (1993). Peahens Lay More Eggs for Peacocks with Larger Trains. Biol. Sci..

[85] Colegrave N., Kotiaho J.S., Tomkins J.L., Sakare J. (2002). Mate choice or polyandry: Reconciling genetic compatibility and good genes sexual selection. Evol. Ecol. Res..

[86] Wilson A.J., Nussey D.H. (2010). What is individual quality? An evolutionary perspective. Trends Ecol. Evol..

[87] Botero-Delgadillo E., Gilsenan C., Mueller J.C., Kempenaers B. (2020). Negative effects of individual heterozygosity on reproductive success in a wild bird population. Mol. Ecol..

[88] Coltman D.W., Slate J. (2003). Microsatellite measures of inbreeding: A meta-analysis. Evolution.

[89] Velando A., Barros Á., Morán P. (2015). Heterozygosity-fitness correlations in a declining seabird population. Mol. Ecol..

[90] Bichet C., Vedder O., Sauer-Gürth H., Becker P.H., Wink M., Bouwhuis S. (2019). Contrasting heterozygosity-fitness correlations across life in a long-lived seabird. Mol. Ecol..

[91] Seddon N., Amos W., Mulder R.A., Tobias J.A. (2004). Male heterozygosity predicts territory size, song structure and reproductive success in a cooperatively breeding bird. Proc. R. Soc. B Biol. Sci..

[92] Ryder T.B., Tori W., Blake J., Loiselle B.A., Parker P. (2009). Mate choice for genetic quality: A test of the heterozygosity and compatibility hypotheses in a lek-breeding bird. Behav. Ecol..

[93] Oh K.P., Badyaev A.V. (2006). Adaptive genetic complementarity in mate choice coexists with selection for elaborate sexual traits. Proc. R. Soc. B Biol. Sci..

[94] Arct A., Drobniak S.M., Mellinger S., Gustafsson L., Cichoń M. (2019). Parental genetic similarity and offspring performance in blue tits in relation to brood size manipulation. Ecol. Evol..

[95] Apanius V., Penn D., Slev P.R., Ruff L.R., Potts W.K. (1997). The nature of selection on the major histocompatibility complex. Crit. Rev. Immunol..

[96] Kokko H., Brooks R., McNamara J.M., Houston A.I. (2002). The sexual selection continuum. Proc. R. Soc. B Biol. Sci..

[97] Brooks R. (2001). Can older males deliver the good genes?. Trends Ecol. Evol..

